# Non-inferiority of low-dose compared to standard high-dose calcium supplementation in pregnancy: study protocol for two randomized, parallel group, non-inferiority trials in India and Tanzania

**DOI:** 10.1186/s13063-021-05811-7

**Published:** 2021-11-24

**Authors:** Pratibha Dwarkanath, Alfa Muhihi, Christopher R. Sudfeld, Shobha Rani, Christopher P. Duggan, Mary M. Sando, Blair J. Wylie, Ryan Fernandez, Shabani Kinyogoli, Cristina Munk, Nandita Perumal, John Michael Raj, Nirmala Buggi, Ndeniria Swai, Tinku Thomas, Molin Wang, Anura V. Kurpad, Honorati Masanja, Andreas B. Pembe, Wafaie W. Fawzi

**Affiliations:** 1 Division of Nutrition, St. John’s Research Institute, Bangalore, India; 2 Africa Academy for Public Health, Dar es Salaam, Tanzania; 3 Department of Global Health and Population, Harvard T.H. Chan School of Public Health, Boston, Massachusetts USA; 4 Department of Nutrition, Harvard T.H. Chan School of Public Health, Boston, Massachusetts USA; 5 Department of Health and Family Welfare, Bruhat Bengaluru Mahanagar Palike, Bangalore, India; 6 Division of Gastroenterology, Hepatology, and Nutrition, Boston Children’s Hospital and Harvard Medical School, Boston, Massachusetts USA; 7 Division of Maternal-Fetal Medicine, Department of Obstetrics & Gynecology, Beth Israel Deaconess Medical Center, Harvard Medical School, Boston, Massachusetts USA; 8 Division of Medical Informatics, St. John’s Research Institute, Bangalore, India; 9 Department of Biostatistics, St. John’s Research Medical College, Bangalore, India; 10 Dar es Salaam Medical Office of Health, Dar es Salaam, Tanzania; 11 Department of Epidemiology, Harvard T.H. Chan School of Public Health, Boston, Massachusetts USA; 12 Department of Biostatistics, Harvard T.H. Chan School of Public Health, Boston, Massachusetts USA; 13 Department of Physiology & Nutrition, St. John’s Medical College & St. John’s Research Institute, Bangalore, India; 14 Ifakara Health Institute, Dar es Salaam, Tanzania; 15 Department of Obstetrics and Gynaecology, Muhimbili University of Health and Allied Sciences, Dar es Salaam, Tanzania

**Keywords:** Calcium, Dietary supplements, Pregnancy, Pregnancy complications, Pre-eclampsia, Pregnancy-induced hypertension, Preterm birth, Non-inferiority trial

## Abstract

**Background:**

Hypertensive disorders of pregnancy are important causes of maternal morbidity and mortality, as well as preterm birth, the leading cause of death for children under 5 years globally. The World Health Organization currently recommends that pregnant women receive high-dose calcium supplementation (1500–2000 mg elemental calcium) for prevention of preeclampsia in populations with low dietary calcium intake. Trials of low-dose calcium supplementation (< 1000 mg elemental calcium/day) during pregnancy have also shown similar reductions in the risk of preeclampsia; however, no trials to date have directly compared low-dose to the standard high-dose calcium supplementation. Our objective is to assess the non-inferiority of low-dose as compared to standard high-dose calcium supplementation in pregnancy.

**Methods/design:**

We will conduct two independent trials in Bangalore, India (*n* = 11,000 pregnancies), and Dar es Salaam, Tanzania (*n* = 11,000 pregnancies). The trial designs are individually randomized, parallel group, quadruple-blind, non-inferiority trials of low-dose calcium supplementation (500 mg elemental calcium/day) as compared to standard high-dose calcium supplementation (1500 mg elemental calcium/day) among nulliparous pregnant women. Pregnant women will be enrolled in the trial before 20 weeks of gestation and will receive the randomized calcium regimen from randomization until the time of delivery. The co-primary outcomes are (i) preeclampsia and (ii) preterm birth; we will test non-inferiority of the primary outcomes for low-dose as compared to the standard high-dose supplementation regimen in each trial. The trials’ secondary outcomes include gestational hypertension, severe features of preeclampsia, pregnancy-related death, third trimester severe anemia, fetal death, stillbirth, low birthweight, small-for-gestational age birth, and infant death.

**Discussion:**

The trials will provide causal evidence on the non-inferiority of low-dose as compared to the standard high-dose supplementation in India and Tanzania. A single tablet, low-dose calcium supplementation regimen may improve individual-level adherence, reduce programmatic costs, and ultimately expand implementation of routine calcium supplementation in pregnancy in populations with low dietary calcium intake.

**Trial registration:**

ClinicalTrials.gov identifier: NCT03350516; registered on 22 November 2018. Clinical Trials Registry—India identifier: CTRI/2018/02/012119; registered on 23 February 2018.

Tanzania Medicines and Medical Devices Authority Trials Registry identifier: TFDA0018/CTR/0010/5; registered on 20 December 2018.

**Supplementary Information:**

The online version contains supplementary material available at 10.1186/s13063-021-05811-7.

## Background

Hypertensive disorders of pregnancy (HDP), including chronic hypertension, gestational hypertension, preeclampsia, eclampsia, and preeclampsia superimposed on chronic hypertension are among the leading causes of maternal and perinatal morbidity and mortality [1–3]. It is estimated that approximately 15% global maternal deaths (~ 30,000 deaths per year) are attributed to hypertensive disorders of pregnancy and that 2–8% of all pregnancies are complicated by preeclampsia with variation by country and context [3–5]. Further, HDP are well-established risk factors for preterm birth which is now the leading cause of death for children under 5 years globally [6–8]. Therefore, addressing the large burden of hypertensive disorders of pregnancy will be essential to reach global maternal and child health goals.

Calcium supplementation in pregnancy has been established as an intervention to reduce the incidence of preeclampsia and preterm birth in populations with low dietary calcium intake [9, 10]. The most recent Cochrane Review meta-analyzed 13 randomized controlled trials of high-dose prenatal calcium supplementation (≥ 1000 mg elemental calcium/day) that included over 15,000 pregnancies and determined that calcium supplementation approximately halved the risk of preeclampsia as compared to placebo (relative risk (RR): 0.45, 95% confidence interval (CI) 0.31 to 0.65) and that the beneficial effects appeared to be larger in populations with low calcium diets [9]. High-dose calcium supplementation also reduced the risk of the composite outcome of maternal death or severe morbidity (RR 0.80, 95% CI 0.66 to 0.98) and reduced the risk of preterm birth (RR 0.76, 95% CI 0.60 to 0.97) [9].

The World Health Organization (WHO) currently recommends that pregnant women receive daily high-dose calcium supplementation (1500–2000 mg oral elemental calcium) for prevention of preeclampsia in populations with low dietary calcium intake [10]. The WHO suggested dosing scheme for calcium supplementation recommends dividing the total dose into three doses that are preferably taken at mealtimes. Further, WHO notes that due to potential negative interactions between iron-containing supplements and calcium supplements, that these two mineral supplements preferably be taken several hours apart [10]. Despite proven efficacy and the WHO recommendation, calcium supplementation in pregnancy is not currently implemented in many low and middle-income countries (LMIC). Two important barriers to implementation of calcium supplementation in pregnancy are the complexity of the dosing scheme and the cost (estimated to be $11.50 per pregnancy for a 6-month supply) of high-dose calcium supplementation regimens [10–13].

The most recent Cochrane review also identified twelve randomized trials of low-dose calcium supplementation (< 1000 mg elemental calcium/day) that included a total of ~ 2300 pregnant women [9]. Low-dose calcium supplementation (with most trials using 500 mg/day) significantly reduced the risk of preeclampsia as compared to placebo (RR 0.38, 95% CI 0.28 to 0.52), and the magnitude of the effect was similar to the effect of high-dose calcium supplementation as compared to placebo [9]. There was no statistically significant effect of low-dose calcium supplementation on preterm birth (RR 0.83, 95% CI 0.34–2.03); however, statistical power was limited, and the wide confidence intervals are consistent with a similar effect as the high-dose calcium supplementation compared to placebo. As a result, it is possible that low-dose and high-dose calcium supplementation may have similar beneficial effects on preeclampsia, preterm birth, and other maternal and child health outcomes. If a once daily low-dose calcium supplementation regimen in pregnancy can provide non-inferior benefits on preeclampsia and preterm birth compared to the recommended high-dose calcium supplementation with a three-dose daily scheme, it may help overcome adherence concerns and barriers for programs to implement routine calcium supplementation in pregnancy.

Our objective is to generate causal evidence for decision-making on the non-inferiority of low-dose as compared to the standard high-dose calcium supplementation for pregnant women in populations with low dietary calcium intake. We will conduct two individually randomized, parallel group, non-inferiority trials of low-dose 500 mg calcium supplementation as compared to standard high-dose 1500 mg calcium supplementation among nulliparous pregnant women in Bangalore, India and Dar es Salaam, Tanzania. The co-primary outcomes are preeclampsia and preterm birth. We will conduct two independent trials in Tanzania and India due to potential differences in dietary calcium intake, the risk and etiology of preeclampsia and preterm birth, and other factors that vary by setting that may result in differences in the non-inferiority of low-dose calcium supplementation. Further, vitamin D is recommended to be provided in combination with calcium supplements in pregnancy in India but not Tanzania. It is also important to provide comparable evidence in the context of South Asia and sub-Saharan Africa to better inform global decision-making on low-dose versus high-dose calcium supplementation during pregnancy.

## Methods

### Study design

We will conduct two independent trials in India and Tanzania. Each will be an individually randomized, parallel group, non-inferiority trial of low-dose 500 mg calcium supplementation versus standard high-dose 1500 mg calcium supplementation for nulliparous pregnant women. The eligibility criteria, intervention, follow-up methods, and outcome definitions have been unified between the two trials; however, each trial will be conducted, analyzed, and reported separately. The first participant was enrolled in the India trial on November 30, 2018, and follow-up data collection is planned to continue through August 2022. The first participant was enrolled in the Tanzania trial on March 22, 2019, and follow-up data collection is planned to continue through November 2022. This trial protocol was written following the Standard Protocol Items: Recommendations for Interventional trials (SPIRIT) checklist (see Additional file 1).

### Study settings

The trial in India (clinicaltrials.gov identifier: NCT03350516; Clinical Trials Registry—India identifier: CTRI/2018/02/012119) will be conducted in urban Bengaluru at 11 health facilities that provide antenatal care within the Greater Bengaluru Metropolitan Corporation system (locally referred to as the Bruhat Bengaluru Mahanagar Palike (BBMP)) (clinicaltrials.gov identifier: NCT03350516 and Clinical trials registry—India identifier: CTRI/2018/02/012119). The trial in Tanzania will be conducted in urban Dar es Salaam at 6 health facilities that provide antenatal care (clinicaltrials.gov identifier: NCT03350516 and Tanzania Medicines and Medical Devices Authority Trials Registry identifier: TFDA0018/CTR/0010/5).

### Eligibility criteria and recruitment

**Fig. 1:**
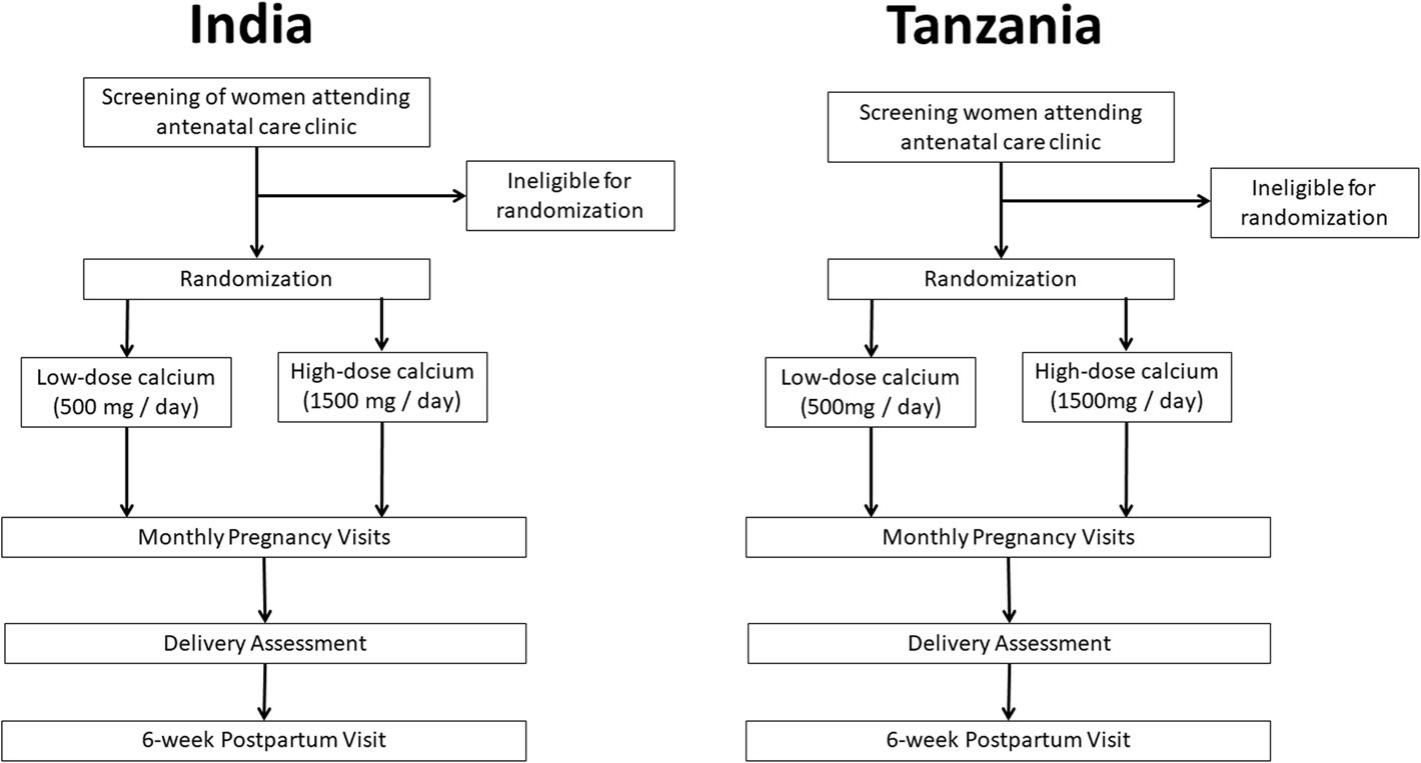
Trial flow diagram for the India and Tanzania trials

The trial flow diagram is presented in Fig. 1. The India and Tanzania trials have the same eligibility criteria for participant enrollment. The inclusion criteria are (a) nulliparous pregnancy, (b) attend first antenatal care visit at the study clinic, (c) less than 20 weeks gestation based on last menstrual period at the time of randomization, (d) ≥ 18 years old, (e) intend to stay in study area until 6 weeks postpartum, and (f) provide written informed consent. Pregnant women will be excluded if they have a (a) history, signs, or symptoms of nephrolithiasis, (b) prior diagnosis of parathyroid disorder or thyroidectomy, or (c) disease that requires digoxin, phenytoin, or tetracycline therapy. Trained research staff will assess eligibility criteria for all participants presenting to the antenatal care clinic and ask potential participants for written informed consent for trial enrollment. Participants will also be asked for their written consent for use of their data and biological specimens (if applicable) in ancillary studies. During the conduct of the trial, the investigators will monitor monthly enrollment targets for the trials and may consider including additional study clinics to increase enrollment if recruitment is slower than expected.

### Interventions

The India and Tanzania trials will assess the non-inferiority of a low-dose calcium supplementation regimen (500 mg elemental calcium/day) as compared to the standard high-dose calcium supplementation regimen (1500 mg elemental calcium/day). The trial regimens will be taken by pregnant women from randomization to the time of delivery (no postnatal supplementation). The standard high-dose 1500 mg calcium supplementation regimen was selected to align with WHO recommendations [10]. The low-dose calcium supplementation regimen was selected to align with low-dose calcium supplementation regimens used in prior low-dose calcium trials [9]. Both randomized groups will take three regimen tablets per day to have identical pill burden in both randomized groups. The trial regimen is manufactured by Influx Healthcare (Maharashtra, India).

Women in both randomized groups will receive a regimen box containing a 35-day supply of trial regimen in blister packs. Each blister pack will contain a 7-day supply of trial regimen (7 × 3 tablets per blister pack) with columns indicating morning, midday, and evening tablets. A diagram of the regimen blister pack for each trial is presented in Additional file 2. Participants in both groups will be counseled to take one trial regimen tablet in the morning, one in the midday, and one in the evening with meals and to take iron-containing prenatal supplements provided as standard of care several hours apart from the calcium trial regimen.

Standard high-dose calcium group (1500 mg elemental calcium/day): Pregnant women will be provided and counseled to take three regimen tablets daily with each containing 500 mg elemental calcium as calcium carbonate supplements daily (total of 1500 mg elemental calcium). In India, daily vitamin D_3_ supplementation (250 IU) is recommended to be taken with calcium supplements and therefore each tablet in the standard high-dose calcium group will also contain 83.3 IU of vitamin D_3_ for a total of 250 IU vitamin D_3_ daily [14]. In Tanzania, the supplements in the standard high-dose calcium group will not contain vitamin D_3_.

Low-dose calcium group (500 mg elemental calcium/day): pregnant women will be provided and counseled to take three regimen tablets each day with the first morning pill containing 500 mg elemental calcium as calcium carbonate, while the second (midday) and third (evening) tablets will be placebo supplements (total of 500 mg elemental calcium per day). The placebo tablets will be identical in all ways as compared to the calcium supplements (appearance, color, shape, size, weight, taste). In India, each of the three daily regimen tablets in the low-dose calcium group will also contain 83.3 IU of vitamin D_3_ for a total of 250 IU vitamin D_3_/day. Therefore, the daily vitamin D_3_ dose will be identical between standard high-dose and low-dose calcium groups in India. In Tanzania, the supplements in the low-dose calcium group will also not contain vitamin D_3_.

Multiple strategies will be used to improve adherence to interventions in India and Tanzania. At both sites, research staff will take a pill count at each study visit by counting the number of unconsumed tablets returned in the regimen blister packs. Study staff will counsel participants on taking the trial regimen and provide advice on overcoming barriers. At the India site, community health workers (accredited social health activists (ASHAs)) will also be engaged to promote regimen adherence during their routine home visits. At the Tanzania site, biweekly text message reminders for adherence and upcoming study visits will be sent. At both the India and Tanzania trial sites, reminder telephone calls will also be made prior participants’ scheduled visits by the research team and if the subject anticipates that she will not be able to attend the clinic visit or does not attend the clinic visit as planned, a home visit will be made by research staff. The research staff will provide the regimen, take pill counts, and conduct clinical assessments (including blood pressure and proteinuria) at home visits.

Participants may withdraw from the study at any time. In addition, study investigators or study physicians can decide that a participant should not continue to take the trial regimen or participate in the study if there are safety concerns or if continued participation does not appear in the best interest of the participant.

### Concomitant care provided during the trial

At each trial site, all participants will be provided with standard of care throughout follow-up according to the national guidelines for antenatal care for India and Tanzania. Standard of care provided by clinic staff in Tanzania includes provision of daily iron-folic acid (IFA) supplements containing 60 mg iron and 0.4 mg folic acid. In India, pregnant women receive daily supplements containing 60 mg of iron throughout pregnancy and pregnant women less than 14 weeks of gestation are initially given daily 5 mg of folic acid until the end of the first trimester followed by daily 0.4 mg folic acid until delivery. Participants will have access to study clinics for post-trial care through the routine health system.

### Assignment of interventions: allocation and blinding

In both trials, pregnant women who consent for enrollment will be individually randomized in a 1:1 ratio to either the (i) low-dose 500 mg calcium supplementation group or (ii) the standard high-dose 1500 mg calcium supplementation. The allocation sequence was generated by a statistician in Boston for each trial separately (India and Tanzania) using a computer-generated randomization list of 11,000 individuals with block randomization, stratified by study clinic. Two non-study staff hold the randomization list codes until completion of primary analyses or until requested by the Data and Safety Monitoring Board (DSMB). An independent study pharmacist in each country will privately prepare the 500 mg and 1500 mg regimen boxes with participant IDs based on the randomization list. At the randomization visit, research staff will assign pregnant women who are eligible and consent for trial randomization to the next available participant ID which corresponds to a set of pre-labeled regimen boxes. The trial statistician and data analysts will also be blinded to treatment allocation for the primary statistical analysis. As a result, the trial is quadruple blind with all trial participants, research staff, and care providers, Investigators, outcome assessors, and trials statistician and data analysts being unable to determine the allocated trial arm for any trial participant or identify trial participants who are on the same trial regimen. Further, the randomization procedures ensure complete allocation concealment. The DSMB, Institutional Review Boards (IRBs), attending physicians, and Principal Investigators may request to unblind a participant(s) allocated trial group during the trial for safety reasons.

### Sample size

The sample size for each trial will be 11,000 participants. Statistical power for the primary outcomes of preeclampsia and preterm birth was calculated using formulas for binomial outcomes in non-inferiority trials [15]. Power calculations assumed 1:1 randomization, a one-sided test with a type I error rate (α) of 0.05, and a 10% loss-to-follow-up rate (including pregnancy loss for preterm outcome). The non-inferiority margin for each primary outcome was selected by the trials’ Technical Advisory Group. The non-inferiority margin for preeclampsia was set at a relative risk of 1.54 for low-dose calcium as compared to the standard high-dose calcium based on the lower bound of the 90% confidence interval for the effect of standard high-dose calcium versus placebo on preeclampsia [9, 16]. The non-inferiority margin for preterm birth was set at a relative risk of 1.16 for low-dose calcium as compared to the standard high-dose calcium regimen to maintain 50% of the effect based on the point estimate of the relative risk for the effect of standard high-dose calcium versus placebo on preterm birth (relative risk = 1.32) [9, 16]. The exact cumulative incidence of preeclampsia and proportion of preterm birth in the standard high-dose calcium arm was not known for the India and Tanzania sites, and therefore, power calculations were presented for a plausible range of cumulative incidence of preeclampsia of 1.5 to 5% and a proportion of preterm birth of 10.0 to 13.5%. Table 1 presents statistical power in each trial for the plausible range of preeclampsia and preterm birth in the standard high-dose calcium group. For preeclampsia, each trial will have ~ 95% power if the cumulative incidence of preeclampsia is as low as 1.5% in the standard high-dose group and > 99.9% if the incidence is high at 5%. For preterm birth, each trial will have 84.3% power if the cumulative incidence is as low as 10% in the standard high-dose group and > 99.9% if the incidence is as high as 13.5%.

**Table 1:** Statistical power for primary outcomes of preeclampsia and preterm birth for each trial site based on a one-sided test with a type I error rate of 0.05 (India = 11,000 participants; Tanzania 11,000 participants)

		Cumulative incidence of preeclampsia in standard high-dose calcium group (1500 mg/day)		
		1.5%	3%	5%
**Statistical power for preeclampsia**	Noninferiority margin—relative risk of 1.54	**95.3%**	**99.9%**	**> 99.9%**
		Proportion of preterm (< 37 weeks) in standard high-dose calcium group (1500 mg/day)		
		10.0%	12.5%	13.5%
**Statistical power for preterm birth**	Noninferiority margin—relative risk of 1.16	**84.3%**	**91.4%**	**93.3%**

### Participant timeline

**Fig. 2:**
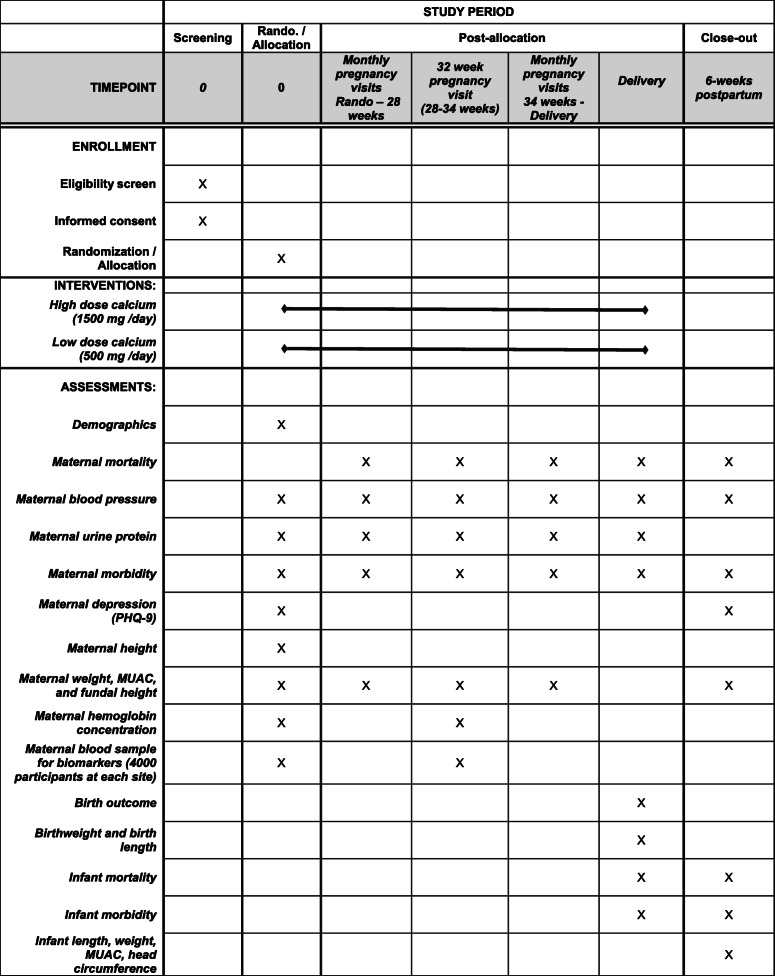
Schedule of enrollment, interventions, and assessments (SPIRIT figure)

The schedule of trial enrollment, interventions, and assessments is presented in Fig. 2. Participant study visits will include a randomization visit, pregnancy study visits every 4 weeks until delivery, a delivery visit, and a postnatal visit scheduled after 42 days postpartum (discharge). Mothers who have a fetal loss or a newborn death will be followed up after 42 days to ascertain maternal mortality. Participant retention will be promoted at clinic visits and through phone calls and home visits.

### Pregnancy study visits

All pregnant women will have a research visit at the time of randomization and every 4 weeks during pregnancy until the time of delivery. Starting at 32 weeks gestation, all women will also receive weekly phone calls and, if possible, will be asked to call the study team around the time of labor. All women will receive an ultrasound for gestational dating around the time of the randomization visit or shortly thereafter. In India, the ultrasounds will be performed by trained and authorized radiologists/sonographers using the ultrasound machines available at each facility. For quality control, Dr. Babu Phillips of the St. John’s Medical College Hospital will review images for each sonographer at the study sites every 6 months. In Tanzania, trained nurses will conduct ultrasounds with General Electronic Vscan Access portable ultrasound machines. All ultrasound images for the Tanzania site will be electronically stored and a random 10% for the duration of the trial will be assessed for quality by Dr. Blair Wylie and Ms. Adair Kendall (sonographer) of the Beth Israel Deaconess Medical Center in Boston and feedback will be given to sonographers in Tanzania for quality improvement.

At all prenatal visits, study physicians will perform a full clinical examination, and treat all comorbidities in accordance with standard of care. Blood pressure will be assessed at randomization and at each pregnancy study visit. Electronic blood pressure monitors will be used in India (Omron HEM-7130, Kyoto, Japan) and Tanzania (Omron HEM-7131, Kyoto, Japan). Blood pressure assessment will be standardized by having all participants rest in a seated position for 5 min, placing the cuff on the left arm at the level of the heart and taking two blood pressure measurements (systolic and diastolic) at a 30-s interval. The electronic blood pressure machines will be calibrated once a month against a mercury sphygmomanometer; the electronic blood pressure machine will be discontinued from use in the study if the measured difference from the mercury sphygmomanometer is greater than 5 mmHg for systolic or diastolic blood pressure. Participants will be asked by study staff to provide urine at the randomization and each pregnancy follow-up visits, and the presence of protein will be assessed with a dipstick. Research staff will collect maternal height with calibrated stadiometers to the nearest 0.1 cm at the randomization visit. At randomization and all follow-up visits, maternal weight will be taken on digital weighing scales to the nearest 0.01 kg and the mid-upper arm circumference (MUAC) will be taken with a measuring tape to the nearest 0.1 cm. The weighing scales will be calibrated each day using certified 5, 10, and 20 kg weights. Pregnant women will have a finger-prick blood sample collected at randomization and at 32 weeks gestation for assessment of hemoglobin concentration (HemoCue Hb 301 in India and HemoCue Hb 201 in Tanzania, HemoCue AB, Ängelholm, Sweden). Hemocue machines will be calibrated each day using the tri-level control solutions provided by the manufacturer. Maternal dietary intake will be assessed with an open-ended 24-h diet recall conducted once during each trimester of pregnancy. Maternal depression will be assessed with the Patient Health Questionnaire (PHQ)-9 at randomization and at the 6 week postpartum visit [17]. We will also seek consent to collect 8 ml of venous blood specimen at randomization and 32 weeks gestation from participants enrolled at each site starting in January 2021 to understand the pathophysiology of hypertensive diseases of pregnancy and preterm birth using a nested case-cohort study design. Blood specimen will be collected in two vacutainers (4 ml each) with the anticoagulant ethylenediamine tetraacetic acid (EDTA) and temporarily stored in a cool box with icepacks until they are transported to a central laboratory at each site for processing within 4 h of collection in order to maintain the cold chain. At the laboratory, trained study technicians will centrifuge the blood specimen tubes to separate the plasma, which will then be aliquoted into multiple, prelabelled cryovials for long-term storage at − 80 °C freezers. Serum samples will be tested for biomarkers of calcium metabolism, pro- and anti-angiogenic profile, and other potential biomarkers related to preeclampsia and preterm birth. The selection of biomarkers and laboratory methods will be finalized in collaboration with the trials’ Technical Advisory Group in mid-2022 and will account for emerging evidence on the pathophysiology of preeclampsia and preterm birth. Study participants who consent for blood collection will also be asked for consent for long-term storage of serum samples and dried blood spots and their use in ancillary studies.

### Labor and delivery study visit

Study staff will be available throughout the day and night to attend labor and delivery of participants. Participants who deliver outside of the study area will be reached by phone by the research staff to obtain relevant information from the mother and/or facility records. Clinic records and interviews with the clinic staff and participants will be used to ascertain the duration of each stage of labor, labor complications, and Apgar scores at 1 and 5 min for the newborns. Maternal blood pressure and proteinuria will be assessed by clinic or study staff or collected from clinic records. Infant length and weight will also be taken by study staff or recorded from facility records. Infant weight will be measured to the nearest gram using a digital scale and length with the use of a rigid length board with an adjustable foot piece to 1-mm precision. Verbal autopsies will be conducted to ascertain causes of maternal deaths [18].

### Postpartum study visit

Women or women/infant pairs will have a study visit at 6 weeks postpartum (42 days) and will be discharged from the trial after completion of this visit. At the postpartum visit, research staff will assess maternal and infant morbidity and hospitalization history. Nurses will also collect weight and, blood pressure from mothers. Study nurses will assess infant feeding, infant weight with a digital infant balance scale to the nearest gram and length to 1-mm precision with a rigid length board with an adjustable foot piece. Study nurses will measure infant head circumference and mid-upper arm circumference (MUAC) with flexible measuring tape. All infant anthropometric measurements will be recorded in duplicate. Verbal autopsies will be conducted to ascertain causes of infant deaths [18].

### Data management

All data will be entered into independent electronic data capture systems in Tanzania and India, and the program will have in-built skip patterns and range check validations for each variable. All identifiable electronic data will remain in India and Tanzania and will be stored on secure local servers that are only accessible by the respective study data teams and Investigators.

## Outcomes

A summary table of the primary outcomes and definitions is presented in Table 2. The primary outcomes for the trials are (i) preeclampsia and (ii) preterm birth. Preeclampsia will be defined by the presence of both gestational hypertension and gestational proteinuria among participants without chronic hypertension, gestational proteinuria among participants with chronic hypertension (superimposed preeclampsia), clinical diagnosis of preeclampsia, or development of severe features with or without proteinuria [1, 21]. A primary outcomes committee composed of the trial investigators and led by a maternal-fetal medicine physician (BJW) will review all potential preeclampsia events blinded to randomized treatment group using established criteria. The preeclampsia event will be confirmed if the majority of the committee members are in agreement with a preeclampsia diagnosis.

**Table 2:** Summary of primary and secondary outcome definitions and analytic populations

Primary outcomes	Definition
Preeclampsia	Gestational hypertension and gestational proteinuria among participants without chronic hypertension; or, gestational proteinuria among participants with chronic hypertension (superimposed preeclampsia); or, clinical diagnosis of preeclampsia by managing clinical team; or, development of severe features of preeclampsia with or without proteinuria
Preterm birth	Live birth < 37 weeks completed weeks gestation
**Secondary outcomes**	**Definition**
Gestational hypertension	A systolic blood pressure ≥ 140 mm Hg on two occasions at least 1 h apart; or, diastolic blood pressure ≥ 90 mm Hg on two occasions at least 1 h apart; or, severe gestational hypertension in pregnancy among participants without chronic hypertension, defined in the antenatal period as systolic blood pressure ≥ 160 mmHg or a diastolic blood pressure ≥ 110 mmHg on two occasions at least 1 min apart after 20 weeks gestation and defined at the time of labor/delivery as requiring only one systolic blood pressure ≥ 160 mmHg or a diastolic blood pressure ≥ 110 mmHg. Note: Chronic hypertension defined by a clinical diagnosis of chronic hypertension (with or without need for medication) or systolic blood pressure ≥ 140 mm Hg on two occasions at least 1 h apart or diastolic blood pressure ≥ 90 mm Hg on two occasions at least 1 h apart before 20 weeks’ gestation.
Severe features of preeclampsia	The following will be defined as severe features of preeclampsia: • Severe gestational hypertension (with or without proteinuria), or • Eclampsia, or • Evidence of end organ dysfunction by laboratory criteria, or • Clinical diagnosis of HELLP syndrome (hemolysis, elevated liver function, low platelets), or • Development of pulmonary edema, or • New onset CNS or visual symptoms
Pregnancy-related death within 42 days of termination of pregnancy	Death of a woman while pregnant or within 42 days of termination of pregnancy, irrespective of the cause of death
Third trimester severe anemia (targeted safety outcome)	Hemoglobin concentration < 7.0 g/dL [19]
Fetal death	A product of human conception, irrespective of the duration of the pregnancy, which, after expulsion or extraction, does *not* breath or show any other evidence of life such as beating of the heart, pulsation of the umbilical cord, or definite movement of voluntary muscles, whether or not the umbilical cord has been cut or the placenta is attached [20]
Stillbirth	Fetal death ≥ 28 weeks gestation
Low birthweight	Live birth weighing < 2500 g
Small-for-gestational age birth	Live birth with size-for-gestational age < 10th percentile on the INTERGROWTH-21st standard
Infant death < 42 days	Death of a live birth during the first 42 completed days of life (6 weeks postpartum)

The co-primary outcome of preterm birth will be defined as a live birth born < 37 completed weeks gestation. We will use the best obstetric estimate (BOE) approach for gestational age dating which combines information from date of last menstrual period (LMP) and fetal biometric measures from ultrasound assessment. Ultrasound-based gestational age will be assessed using INTERGROWTH-21st equations for gestational age assessment for crown-rump length, head circumference, and femur length, or the Hadlock equations for biparietal diameter and femur length, using a hierarchical approach (Additional file 3) [22, 23]. First and second trimester fetal biometric measures will be used when possible. Ultrasound-based gestational age will be used if the estimated date of delivery (EDD) based on fetal biometric measures differs from LMP-based EDD according to guidelines established by the Society for Maternal-Fetal Medicine and the American College of Obstetrics and Gynecology (Additional file 3) [24]. For any participant for whom ultrasound scans could not be conducted, are not available, or are deemed to be invalid, LMP-based GA will be used.

The secondary trials outcomes and definitions are presented in Table 2. Third trimester severe anemia (hemoglobin concentration < 7.0 g/dL) was identified as a targeted safety outcome that will be reported as a secondary outcome in the main trial report. All women will have hemoglobin concentrations assessed at 32 weeks gestation. Calcium supplements can reduce absorption of iron and therefore may increase the risk of anemia [10].

## Statistical methods

The India and Tanzania trials will be analyzed independently; however, the outcome definitions and statistical methods will be the same for both trials. An intent-to-treat (ITT) analysis will be the primary approach for all analyses and one-sided tests for non-inferiority with a type I error rate of 0.05 will be used. The ITT analysis for preeclampsia will include all randomized women and the preterm birth ITT analysis will include all livebirths. A complete case analysis will be used in the primary analyses.

For preeclampsia, we will use a log-binomial model with a fixed effect for study clinic to account for the stratified randomization scheme to produce relative risk estimates for the preeclampsia outcome for the low-dose as compared to the standard high-dose calcium supplementation group. The upper bound of the 90% confidence interval of the relative risk for preeclampsia will be compared with the prespecified noninferiority margin to assess non-inferiority. The primary analysis for preeclampsia will be among all randomized participants. We will conduct a sensitivity analysis for preeclampsia excluding pregnancy losses and participants who withdraw before 20 weeks gestation. In addition, we will also construct Kaplan–Meier curves with gestational age as the time metric to visualize the incidence of preeclampsia between randomized groups. We will present relative risks and 90% confidence intervals but will not formally test non-inferiority for any preeclampsia sensitivity analyses.

For preterm birth, a generalized estimating equation (GEE) analysis with the log link, a fixed effect for study clinic and a compound symmetry working correlation matrix to account for correlations due to multiple gestation will be used to construct relative risks for preterm birth among live births between randomized groups. The 90% confidence intervals of the relative risk will be compared with the prespecified non-inferiority margin to assess non-inferiority. We will conduct a sensitivity analysis for preterm birth among singleton pregnancies and will present relative risks and 90% confidence intervals but will not formally test non-inferiority.

We will also conduct a per-protocol analysis for preeclampsia and preterm birth. The per-protocol analysis for preeclampsia will include all participants who had > 75% adherence to the supplementation regimen based on pill count, had a pregnancy ≥ 20 weeks gestation, and had a delivery outcome assessed (excluding withdrawal and loss-to-follow-up in pregnancy). The per-protocol analysis for preterm birth will include all participants who had > 75% adherence to the supplementation regimen and a live birth. We will present relative risks and 90% confidence intervals for the per-protocol analyses but will not formally test non-inferiority.

We will use similar methods for analysis of secondary binomial maternal and infant outcomes (Table 2). We will present relative risks and 95% confidence intervals for secondary outcomes but will not formally test non-inferiority between randomized groups.

## Oversight and monitoring

The trials will be advised by a Technical Advisory Group (TAG) of experts in calcium supplementation in pregnancy, obstetrics and gynecology, and clinical trials that will meet with the study team every 6 months to provide guidance on implementation and evaluation of the trials. The TAG members include the following: Dr. Tahmeed Ahmed (International Centre for Diarrhoeal Disease Research, Bangladesh), Dr. José Belizán (Institute for Clinical Effectiveness and Health Policy (IECS-CONICET), Argentina), Dr. Ana Pilar Betrán (World Health Organization, Switzerland), and Dr. Friday Okonofua (Ondo State University of Medical Sciences, Nigeria). In addition, the TAG set the non-inferiority margins for the primary outcomes of the trials. Dr. Per Ashorn (Tampere University, Finland) and Dr. Scott Evans (George Washington University, USA), who are experts in clinical trials and biostatistics, participated in the TAG to set the non-inferiority margins.

The trials will be overseen by a Data and Safety Monitoring Board (DSMB) that will meet every 6 months to review the trial, assess adverse events and apply stopping rules for benefit, futility, or harm. The DSMB members include Dr. Janet Rich-Edwards (Chair; Brigham & Women’s Hospital, USA), Dr. Nita Bhandari (Society for Applied Studies, India), Dr. Justus Hofmeyr (University of the Witwatersrand and University of Fort Hare, South Africa), Dr. Elia Mmbaga (Muhimbili University of Health and Allied Sciences, Tanzania), and Dr. Paige Williams (Harvard T.H. Chan School of Public Health, USA). The DSMB will review and make decisions on the India and Tanzania trials independently. The DSMB plans to conduct an interim data analysis by blinded study arm for each trial when one-third and two-thirds of deliveries have occurred. The DSMB may request unmasking of the data for either safety or efficacy concerns. For the primary endpoints, the two arms will be compared when 1/3 and 2/3 women delivered, respectively. If the *p* value for a difference in the risk of preeclampsia or preterm births between treatment groups is less than 0.001 (the Haybittle-Peto rule), the DSMB will consider unblinding the trial and stopping will be considered. The DSMB will also monitor all severe adverse events (SAEs) and the targeted safety outcome of third trimester severe anemia at each meeting. If the comparison of the risk of an SAE or third trimester anemia varies significantly between treatment groups at a *p* value of 0.05 or below, the DSMB will assess the need for unblinding and stopping will be considered.

All protocol modifications will be reported to the trial IRBs, TAG, and DSMB prior to implementation. All serious adverse events and adverse events that are at least possibly related to the study regimen or activities are reported to all Institutional Review Boards per regulatory guidelines and to the DSMB.

## Dissemination

We will disseminate the trial findings through academic publications, conference presentations, and at scientific meetings. We will also disseminate our findings to government and non-government stakeholders at the national and local levels in India and Tanzania as well as to international stakeholders.

## Discussion

We will conduct two large, randomized trials to assess the noninferiority of a low-dose calcium supplementation regimen (500 mg/day) for pregnant women as compared to the standard high-dose calcium regimen (1500 mg/day) in India and Tanzania. We expect the trial results to be reported in mid-2023. The trials in India and Tanzania will be analyzed and reported independently as the non-inferiority of low-dose calcium supplementation in pregnancy may differ due to contextual factors and vitamin D is provided in combination with calcium supplements in India but not Tanzania. Further, comparable evidence from the context of South Asia and sub-Saharan Africa will be beneficial for global decision-making on calcium supplementation policies.

It is important to note that our trials have a few limitations by design. First, by design, both the low-dose and standard high-dose calcium supplementation groups will receive 3 tablets per day. As a result, we will assess the biological effect of the calcium regimen since the same number of tablets will be taken by the two trial groups but will not be able to assess potential effects of a single 500 mg regimen pill on adherence and health outcomes. Second, we will only enroll nulliparous pregnant women in our trial due to their high risk of preeclampsia and preterm birth. As a result, it is important to consider the potential generalizability of our findings to multiparous pregnant women; the biological mechanisms leading to the incidence of preeclampsia may be largely shared for multiparous and nulliparous pregnant women, and therefore, our findings may be generalizable to all pregnant women in settings similar to the India and Tanzania trial contexts. Last, we will conduct a non-inferiority trial that will estimate the causal effect of the low-dose as compared to the standard high-dose calcium supplementation regimen and therefore additional studies and implementations evaluations will be needed to evaluate the real-world effectiveness of calcium supplementation programs.

In summary, if low-dose calcium supplementation regimen in pregnancy is non-inferior to standard high-dose supplementation, a single daily dose regimen of calcium containing 500 mg elemental calcium may help address adherence barriers of a three-dose regimen per day as well as cost and logistical barriers to implementation of routine calcium supplementation in pregnancy in health systems. Further, the evidence from the two trials may inform the need to update the WHO calcium supplementation guidelines for pregnant women that recommend 1500–2000 mg calcium per day in populations with low calcium intake.

## Trial status

For the India trial, we report protocol version 2.7 dated August 13, 2020. The India trial enrollment started on 30 November 2018, recruitment is ongoing as of 26 October 2021, and recruitment is expected to be completed in February 2022. For the Tanzania trial, we report protocol version 1.7 dated August 13, 2020. The Tanzania trial enrollment started on 22 March 2019, recruitment is ongoing as of 26 October 2021, and recruitment is expected to be completed in February 2022.

## Supplementary Information

**Additional File 1..** Standard Protocol Items: Reommendations for Interventional Trials (SPIRIT) 2013 Checklist.

**Additional File 2..** Diagram of the India and Tanzania regimen blister packs

**Additional File 3..** Summary of regression equations for gestational age dating and guidelines for redating gestational age based on ultrasound

## Data Availability

The datasets used and/or analyzed during the current study may be made available from the corresponding author on reasonable request and approval from applicable Institutional Review Boards.

## Abbreviations

ASHA: Accredited social health activist
BBMP: Bruhat Bengaluru Mahanagar Palike
CI: Confidence interval
DSMB: Data and Safety Monitoring Board
Hb: Hemoglobin
HDP: Hypertensive disorder of pregnancy
IFA: Iron-folic acid
IRB: Institutional Review Board
IU: International unit
MUAC: Mid-upper arm circumference
TAG: Technical Advisory Group
WHO: World Health Organization
