# Supplementary material for: Non-inferiority of low-dose compared to standard high-dose calcium supplementation in pregnancy: study protocol for two randomized, parallel group, non-inferiority trials in India and Tanzania
Source: Trials. 2021 Nov 24;22:838. doi: 10.1186/s13063-021-05811-7 (PMC8611882; doi:10.1186/s13063-021-05811-7)

Additional File 2. Diagram of the India and Tanzania regimen blister packs

Figure 1. Diagram of the India trial regimen blisterpacks

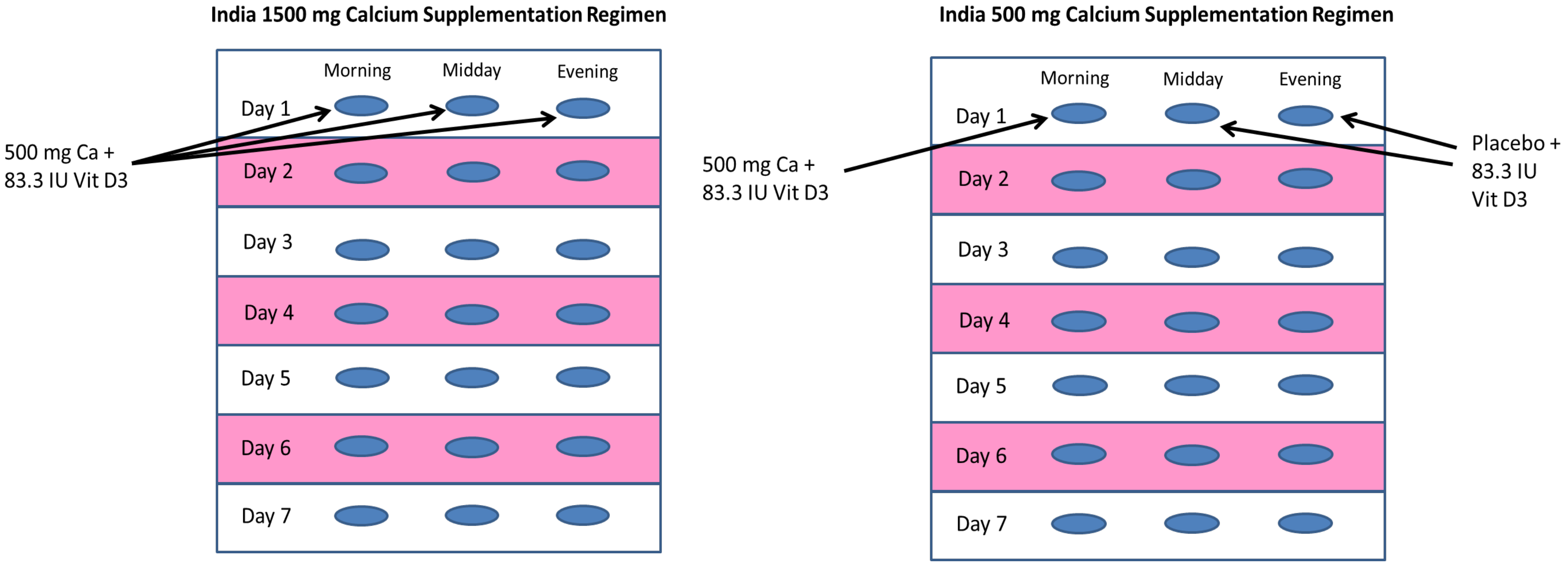

Figure 2. Diagram of the Tanzania trial regimen blister packs

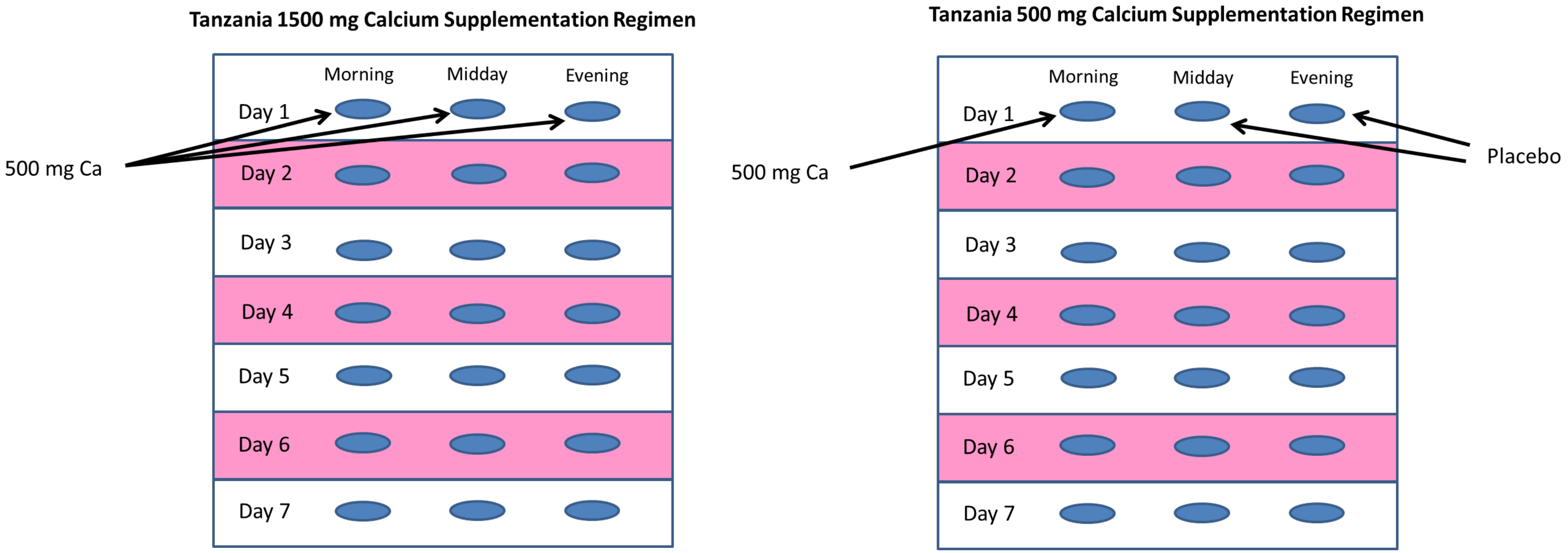

Supplement: Supplementary file 2 — Additional File 2.. Diagram of the India and Tanzania regimen blister packs [file 13063_2021_5811_MOESM2_ESM.pdf]
