# Supplementary material for: Non-inferiority of low-dose compared to standard high-dose calcium supplementation in pregnancy: study protocol for two randomized, parallel group, non-inferiority trials in India and Tanzania
Source: Trials. 2021 Nov 24;22:838. doi: 10.1186/s13063-021-05811-7 (PMC8611882; doi:10.1186/s13063-021-05811-7)
Supplement: Supplementary file 3 — Additional File 3.. Summary of regression equations for gestational age dating and guidelines for redating gestational age based on ultrasound [file 13063_2021_5811_MOESM3_ESM.pdf]

Additional File 3. Summary of regression equations for gestational age dating and guidelines for redating gestational age based on ultrasound

Supplementary Table 1: Regression equations for predicting gestational age from fetal measurements.

Trimester and source of equation	Fetal measurements	Regression equations
$\leq 13^{6/7}$ wks (2)	CRL (mm)	INTERGROWTH-21st<br>Mean of log GA (wk) = 1.4612 + 0.001693*CRL + 0.2332*logCRL<br>SD of log GA = 0.0458114 – 0.00000198*CRL
$14^{0/7}$ to 42 $^{0/7}$ wks (3)<br>(in order of preference, descending priority)	1. HC (mm), FL (mm)	INTERGROWTH-21st<br>GA (wk) =(exp(0.03243*(ln(HC))^2 +0.001644*FL*ln(HC) +3.813))/7; SD=0.04009*GA-1.149;, FL and HC in mm
	2. HC (mm)	INTERGROWTH-21st<br>GA (wk) = (exp(0.05970*(ln(HC^2) +0.000000006409*HC^3 +3.3258))/7; SD=0.6492*(GA*0.01)^3 +2.991; HC in mm
	3. BPD, FL (cm)	Hadlock<br>GA (wk) = 10.50 + 0.197(BPD)(FL) + 0.9500(FL) + 0.7300(BPD)
	4. BPD (cm)	Hadlock<br>GA (wk) = 9.54 + 1.482(BPD) + 0.1676(BPD)^2
	5. FL (cm)	Hadlock<br>GA (wk) = 10.35 + 2.460(FL) + 0.170(FL)^2

CRL = Crown-rump length
FL = femur length
HC = Head circumference

Supplementary Table 2: Society for Maternal-Fetal Medicine and the American College of Obstetrics and Gynecology guideline for redating based on ultrasonography

GA by LMP assessment	Biometric variable	Difference from GA based on LMP
<$8^{6/7}$ wks	CRL	5 days
$9^{0/7}$ to $13^{6/7}$	CRL	7 days
$14^{0/7}$ to $15^{6/7}$	BPD, HC, AC, FL	More than 7 days
$16^{0/7}$ to $21^{6/7}$	BPD, HC, AC, FL	More than 10 days
$22^{0/7}$ to $27^{6/7}$	BPD, HC, AC, FL	More than 14 days
$28^{0/7}$ wk and beyond	BPD, HC, AC, FL	More than 21 days

References:

Ohuma EO, Papageorghiou AT, Villar J, Altman DG. Estimation of gestational age in early pregnancy from crown-rump length when gestational age range is truncated: the case study of INTERGROWTH-21st project. BMC Med Res Methodol. 2013;13:151.

Hadlock FP, Deter RL, Harrist RB, Park SK. Estimating fetal age: computer-assisted analysis of multiple fetal growth parameters. Radiology. 1984;152:497–501.

Committee on Obstetric Practice American Institute of Ultrasound in Medicine Society for Maternal-Fetal Medicine. Method for Estimating Due Date. Committee Opinion 611. Obs Gynecol. 2014;1–4.
